# DEVELOPMENT AND VALIDATION OF A SOCIAL FRAILTY INDEX AMONG OLDER ADULTS IN THE PHILIPPINES

**DOI:** 10.1093/geroni/igae098.2602

**Published:** 2024-12-31

**Authors:** Edina Tan, Hanzhang Xu, Rahul Malhotra, Mark Ryan Paguirigan, Grace Cruz, Yasuhiko Saito, Truls Østbye

**Affiliations:** Duke-NUS Medical School, Singapore, Singapore; Duke University, Durham, North Carolina, United States; Duke-NUS Medical School, Singapore, Singapore; University of the Philippines Diliman, Quezon City, Quezon, Philippines; University of the Philippines Diliman, Quezon City, Quezon, Philippines; Nihon University, Tokyo, Japan; Duke University, Durham, North Carolina, United States

## Abstract

The potential of social frailty, known to be associated with adverse health outcomes among older adults, for risk assessment can be maximized if the development of a scale to measure it considers the sociocultural context in which it will be applied. No social frailty scales have been developed for use in the Philippines, a Southeast Asian country with distinctive sociocultural characteristics such as an emphasis on religious life, family caregiving, and a generally positive view of aging. We developed and validated a Social Frailty Index-Philippines (SFI-Phil), using 4-year mortality as an outcome, for use among older adults in the Philippines. We also evaluated its performance across age, gender, and urban-rural residence. Data on 5153 older adults, aged 60 years and older, from the nationally representative Longitudinal Study of Ageing and Health in the Philippines (LSAHP) was analyzed. Applying LASSO regression on 33 candidate variables selected using a conceptual framework for social frailty, the SFI-Phil included six variables: (1) education, (2) religious activity participation, (3) caring for grandchildren, (4) employment, (5) hanging out with friends and neighbors, and (6) feeling left out. Evaluation of SFI-Phil in a validation sample demonstrated satisfactory accuracy in predicting mortality (C-statistic 0.640, 95% CI: 0.608-0.673). It also performed adequately across demographic strata, although better amongst 60–69 year olds (C-statistic 0.642, 95% CI: 0.606-0.673), males (0.670, 0.650-0.694), and urban residents (0.660, 0.637-0.684). SFI-Phil, developed considering the country’s sociocultural context, can be used to assess social frailty among older adults in the Philippines.

